# Altered mitochondrial bioenergetics are responsible for the delay in Wallerian degeneration observed in neonatal mice

**DOI:** 10.1016/j.nbd.2019.104496

**Published:** 2019-10

**Authors:** Rachel A. Kline, Kosala N. Dissanayake, Maica Llavero Hurtado, Nicolás W. Martínez, Alexander Ahl, Alannah J. Mole, Douglas J. Lamont, Felipe A. Court, Richard R. Ribchester, Thomas M. Wishart, Lyndsay M. Murray

**Affiliations:** a Centre for Discovery Brain Science, University of Edinburgh, Hugh Robson Building, Edinburgh EH8 9XD, UK; b Euan McDonald Centre for Motor Neuron Disease Research, University of Edinburgh, UK; c Centre for Cognitive and Neural Systems, University of Edinburgh, 1 George Square, Edinburgh EH8 9JZ, UK; d The Roslin Institute and Royal (Dick) School of Veterinary Studies, University of Edinburgh, Easter Bush, EH25 9RG, UK; e Center for Integrative Biology, Faculty of Sciences, Universidad Mayor, Santiago, Chile; f Fingerprints Proteomics Facility, Dundee University, Dundee DD1 4HN, United Kingdom; g Geroscience Center for Brain Health and Metabolism, Santiago, Chile; h The Buck Institute for Research on Aging, Novato, CA, United States

**Keywords:** Neurodegeneration, Wallerian, Neonate, Axon degeneration, Neuromuscular junction, NMJ, Proteomics, Mitochondria, 2H3, Neurofilament, AMPK, Adenosine Monophosphate Kinase, DHE, Dihydroethidium, DRG, Dorsal Root Ganglion, ELISA, Enzyme Link Immuno, ETC, Electron Transport Chain, IPA, Ingenuity Pathway Analysis, MPS, Mammalian Physiological Saline, NMJ, Neuromuscular Junction, OXPHOS, Oxidative Phosphorylation, P, Post Natal Day, QFWB, Quantitative Fluorescent Western Blotting, ROS, Reaction Oxygen Species, SV2, Synaptic Vesicle Protein 2, TEAB, Tetraethylammonium Bromide, TMT-QMS, Tandem-Mass Tagging Quantitative Mass Spectrometry, TMT, Tandem Mass Tagging, WD, Wallerian Degeneration

## Abstract

Neurodegenerative and neuromuscular disorders can manifest throughout the lifespan of an individual, from infant to elderly individuals. Axonal and synaptic degeneration are early and critical elements of nearly all human neurodegenerative diseases and neural injury, however the molecular mechanisms which regulate this process are yet to be fully elucidated. Furthermore, how the molecular mechanisms governing degeneration are impacted by the age of the individual is poorly understood. Interestingly, in mice which are under 3 weeks of age, the degeneration of axons and synapses following hypoxic or traumatic injury is significantly slower. This process, known as Wallerian degeneration (WD), is a molecularly and morphologically distinct subtype of neurodegeneration by which axons and synapses undergo distinct fragmentation and death following a range of stimuli. In this study, we first use an *ex-vivo* model of axon injury to confirm the significant delay in WD in neonatal mice. We apply tandem mass-tagging quantitative proteomics to profile both nerve and muscle between P12 and P24 inclusive. Application of unbiased *in silico* workflows to relevant protein identifications highlights a steady elevation in oxidative phosphorylation cascades corresponding to the accelerated degeneration rate. We demonstrate that inhibition of Complex I prevents the axotomy-induced rise in reactive oxygen species and protects axons following injury. Furthermore, we reveal that pharmacological activation of oxidative phosphorylation significantly accelerates degeneration at the neuromuscular junction in neonatal mice. In summary, we reveal dramatic changes in the neuromuscular proteome during post-natal maturation of the neuromuscular system, and demonstrate that endogenous dynamics in mitochondrial bioenergetics during this time window have a functional impact upon regulating the stability of the neuromuscular system.

## Highlights

• There is a decrease in the rate of injury induced Wallerian degeneration in motor axons aged <P12 vs >P24.
• There is a continuous increase in oxidative phosphorylation proteins from P12 to P24 in lumbrical muscle and tibial nerve.
• Inhibition of complex I prevents axon degeneration following injury, and prevents the rise in reactive oxygen species.
• Pharmacological activation of oxidative phosphorylation accelerates axonal and synaptic degeneration in neonatal mice.
• Pharmacological inhibition of complex 1 protects axons from injury induced degeneration.

## Graphical abstract

**Figure:**
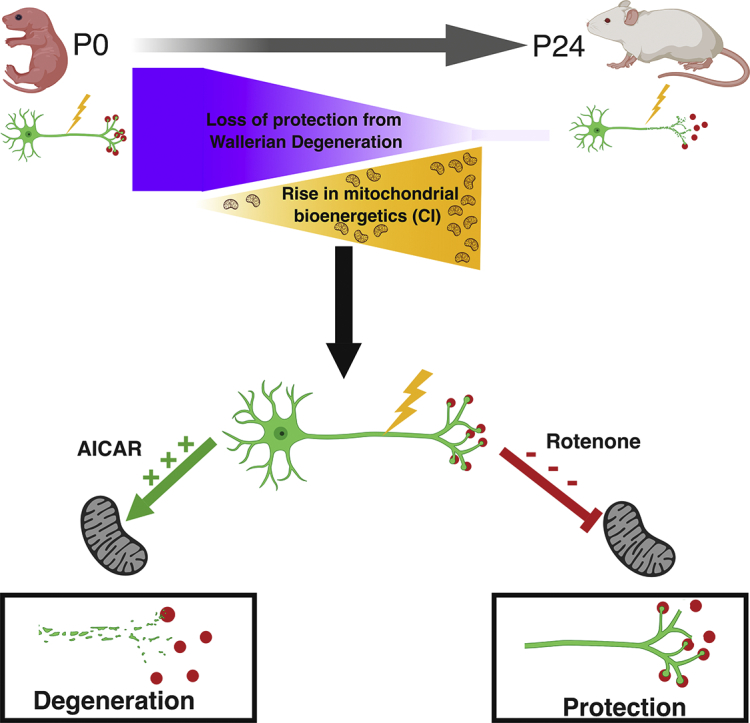

## 1 Introduction

Throughout the lifespan of an individual, neurons are susceptible to degeneration in heritable and spontaneous disease, and following a wide variety of insults, including chemical damage and physical trauma. Neurodegenerative disorders are a primary cause of death in adult and elderly populations, and although specific neurodegenerative disorders in children are considered rare, collectively they have been estimated to account for 28% of admissions to pediatric neurology wards(de Pedro-Cuesta et al., 2015; Dyken and Krawiecki, 1983). Neurodegeneration caused by both pathological and traumatic insult are united by a common vulnerability of the axonal and synaptic compartments of the cell. Although axonal and synaptic degeneration appears to be mechanistically distinct from somatic degeneration, the molecular mechanisms which regulate the vulnerability of the axonal and synaptic compartments of the neuron, and the process by which they degenerate are yet to be fully elucidated. Furthermore, how the molecular mechanisms governing degeneration are impacted by the age of the individual is poorly understood.

Axon and synapses can degenerate in a range of mechanistically distinct manners(Yaron and Schuldiner, 2016). Wallerian degeneration (WD) refers to one of these processes, during which axons and their pre-synaptic terminals undergo rapid fragmentation, degradation and clearance by scavenging macrophages and Schwann Cells (Saxena and Caroni, 2007; Catenaccio et al., 2017). This pattern of degeneration is also observed following trauma to the axon or in diseases that include multiple sclerosis or stroke (Kawachi and Lassmann, 2017; Stoll and Muller, 1999; Wang et al., 2012). We have recently described that the rate of WD following axotomy is significantly slower in neonatal mice compared to adults (Murray et al., 2011). An *ex-vivo* model of hypoxia-reperfusion injury using tissue from adult (P28) mice induced degeneration of 95% of neuromuscular junctions (NMJs) within 24 h. However, the same injury in nerve-muscle preparations from neonatal (P2) mice resulted in loss of motor nerve terminals from only 5% of NMJs. A similar delay in WD following traumatic nerve injury from axotomy was observed in mice aged up to P20. Further investigation into this phenomenon revealed that the delay in the rate of injury-induced NMJ degeneration was progressively lost between P12 and P25. Interestingly, a preconditioning lesion to generate immature NMJs in adult mice (*cf.* (Gillingwater et al., 2002)) failed to mitigate this rapid degeneration response post axotomy in adult mice (Murray et al., 2011). It therefore appears that the regulatory elements responsible for this dramatic increase in the rate of WD at the NMJ arise from the surrounding environment, namely the nerve or muscle, rather than the NMJ itself. Consequently, factors which contribute to the stability and degeneration axonal and synaptic compartments of the cell are developmentally regulated. In order to gain insight into the mechanisms which underlie neurodegeneration, and how they evolve and adapt during the lifespan of an individual, it is important to understand the mechanisms which regulate the delay in axon degeneration in neonatal mice.

While the definitive mechanisms underpinning WD remain unresolved, a number of specific mutations have been shown to profoundly influence its rate, and thus serve as compelling tools to study perturbed WD profiles: Perhaps the best known of these mutations (indeed named for its effect on the process), − Wallerian Degeneration Slow (Wld^s^) - in mice delays axonal degeneration by a factor of ten (Brown et al., 1994; Lyon et al., 1993; Oyebode et al., 2012). This mutation incorporates an in-frame fusion between genes encoding nicotinamide nucleotide adenylyltransferase 1 (Nmnat1) and the N-terminus of ubiquitination factor E4B (Ube4b) (Coleman et al., 1998; Conforti et al., 2000; Wishart et al., 2007). Transgenic expression of the chimeric protein delays axon degeneration following nerve injury in mice, rats, *Drosophila* and zebrafish (Adalbert et al., 2005; Hoopfer et al., 2006; Mack et al., 2001; O'Donnell et al., 2014; Wang et al., 2001a; Wishart et al., 2012). Axonal protection by Wld^S^ protein is also observed following a range of traumatic, chemical and pathological insults, in both the central and peripheral nervous systems (Beirowski et al., 2008; Beirowski et al., 2010; Ferri et al., 2003; Gillingwater et al., 2004; Howell et al., 2007; Meyer zu Horste et al., 2011; Mi et al., 2005; Sajadi et al., 2004; Samsam et al., 2003; Wang et al., 2001b, 2002; Zhu et al., 2014).

Recently, Sterile Alpha and TIR motif-containing 1 (*Sarm1*) was identified as a critical component of the Wallerian Degeneration pathway. Knockout of *Sarm1* in both *Drosophila* and mice phenocopies *Wld*^*S*^ gene expression and protects axons from WD for weeks post axotomy (Osterloh et al., 2012). Studies of Wld^S^ and Sarm1, and the mechanisms by which they exert their protective influence have not only enabled the discovery of key molecular players involved in the degenerative process, but also have cast light upon other factors that may modulate axonal and synaptic protection. Indeed, work on both Wld^s^ and Sarm1 suggest mitochondria have an important role in the progression of WD. This was first suggested as a possibility ten years ago following early proteomic investigations into altered synaptic stability using Wld^s^ (Wishart et al., 2007). Subsequently, Barrientos et al., demonstrated that inhibition of the mitochondrial permeability transition pore (MPTP) could phenocopy the axon protection observed with Wld^s^ (Barrientos et al., 2011). More recently Avery et al. demonstrated that Wld^s^ is indeed likely acting through altered mitochondrial processes such as activity, dynamics and buffering capacity (Avery et al., 2012). Although mitochondria have been strongly implicated in the process of WD (Court and Coleman, 2012), their importance in the process of WD has also been questioned. Specifically, the protective effects of *Wld*^*s*^ were still present in axon depleted of mitochondria (Kitay et al., 2013) and while over expression of Nmnat2 was protective to the axon, it does not appear to colocalise with the mitochondria (Milde et al., 2013). Although changes in energy status and ATP production have been implicated in WD, it has been suggested that this is due to changes in glycolysis, rather than mitochondrial respiration (Godzik and Coleman, 2015). Furthermore, although *Sarm1* knockout or an increase in Nmn levels can protect axons from degeneration, they do no prevent changes in mitochondrial motility or depolarisation (Loreto et al., 2015). The precise involvement of mitochondria in the process of WD remains somewhat controversial, and further work is required to define their involvement and importance in regulating axon degeneration.

In this present study, we hypothesized that the observed acceleration in the rate of synaptic and axonal WD is attributed to dynamic changes in the postnatal nerve and muscle proteome. We utilized an *ex-vivo* model of nerve injury to re-examine the age-dependent observations we reported previously (Murray et al., 2011). This confirmed that delayed NMJ degeneration was progressively lost between ages of P10 to P26. To address the mechanisms, we utilized tandem-mass tagging quantitative mass spectrometry (TMT-QMS) to profile the proteome of both the nerve and muscle at 5 time points between P12 and P24. This resulted in the identification of 7440 and 6079 proteins in nerve and muscle respectively. Following a bioinformatics-based refinement of the data, we employed pathway analysis of the most biologically relevant protein alterations to reveal an up-regulation in molecular networks implicated in oxidative phosphorylation (OXPHOS) and related mitochondrial functions. Finally, we demonstrated that pharmacological up-regulation of basal OXPHOS activity levels accelerated synaptic degeneration, whilst exposure to the Complex I inhibitor rotenone was strongly axoprotective. In summary, this study details post-natal changes in the nerve muscle proteome, and identifies protein changes which can account for the delay in axon degeneration observed in neonatal mice.

## 2 Materials and methods

### 2.1 Animal handling and husbandry

All procedures were performed in adherence with the guidelines set out by the UK Home Office. MCos1 C56BL6/J mice were maintained under Specific and Opportunistic Pathogen-Free conditions in breeding facilities at the University of Edinburgh. All mice were sacrificed by an overdose of anesthetic and cervical dislocation.

### 2.2 Ex-vivo model of nerve injury

Mice ranging from P12 to P24 were sacrificed and immediately dissected to minimize any post-mortem molecular changes. Hind legs were removed and legs were microdissected in mammalian physiological saline (MPS) of following ionic composition (mM): Na^+^ (158.4); K^+^ (5); Ca^2+^ (2); Mg^2+^ (1); Cl^−^ (145); HCO_3_^–^ (24), H_2_PO_4_^−^ (0.4), glucose (5); equilibrated by bubbling in 95%O_2_/5% CO_2_ to pH 7.2–7.4. The deep lumbrical muscles with overlying FDB tendon and muscle attached were dissected, along with the distal nerve stump of the sciatic nerve, including all the nerve branches which innervate the lumbrical muscles. This preparation therefore represented the muscles with attached innervation, wherein the axons have been severed from the cell body. This system therefore reflects an *ex-vivo* model of axon injury. This preparation was pinned to dental wax and incubated for 24 h at 28 °C in oxygenated MPS bubbled continuously with 95/5% O_2_/CO_2_. When specified, 2 mM AICAR (Sigma) was added to oxygenated ringer solution.

### 2.3 Immunocytochemical staining of nerve-muscle explants

Following 24 h incubation *ex-vivo,* nerve-muscle preparations were incubated at room temperature with TRITC-conjugated α-bungarotoxin at a concentration of 5 μg/ml in phosphate buffered saline to label AChR at motor endplates. Preparations were fixed at room temperature for 15 min in 4% paraformaldehyde in PBS, then incubated for 72 h at 4 °C with primary antibodies against synaptic vesicle protein (SV2), and neurofilament (2H3) at a concentration of 1:100 and 1:50 respectively (Developmental Studies Hybridoma Bank, Iowa) then for 24 h at 4 °C in FITC-conjugated IgG anti-mouse secondary antibody (Jackson).

### 2.4 Imaging and analysis

Endplate occupancy was quantified using a Leica DMi8 inverted epifluorescent microscope (10×, 20× and 40× objectives; 0.53, 0.55 and 0.9NA; Leica DMi9 microscope, Leica DFC7000-T camera). Quantification of endplate occupancy was performed blind, and incorporated a minimum of 50 NMJs from at least three distinct fields of view per muscle per mouse (*n* *=* number of muscles, N = number of mice), using the following criteria: fully occupied, *i.e.* complete coverage of the post-synaptic endplate by the branches of the nerve terminal; partially occupied, or vacant, ie. complete withdrawal of the pre-synaptic terminal from the post-synaptic endplate.

Individual statistical tests and *n* numbers used are noted in figure legends. Statistical significance was considered to be *p* ≤ 0.05.

Confocal microscopy was performed using a Nikon A1R^+^ Resonant Scanning System (Nikon) (10× and 40× objectives; 0.3 and 1.3 oil NA; Nikon A1R^+^ microscope; simultaneous image acquisition). 488 and 543 nm laser lines were used for excitation. The resultant confocal *Z*-series produced in NIS Elements 2D Analysis software were exported and merged using public domain Fiji ImageJ software downloadable from https://fiji.sc/.

Figures were created using GNU Image Manipulation Program (GIMP) for Windows.

### 2.5 Sample preparation for LC-MS/MS

Tandem mass tagging and fractionation of extracted samples was performed by the FingerPrints Proteomics facilities at the University of Dundee, to the following protocol:

Protein samples were thawed, trypsinised and desalted at room temperature. 100 μg of desalted tryptic peptides per sample were dissolved in 100 μl of 100 mM tetraethylammonium bromide (TEAB). The 10 different tandem mass tag (TMT) labels comprising the TMT10plex™ kit (Thermo Fisher Scientific) were dissolved in 41 μl anhydrous acetonitrile. Each dissolved label was added to a different sample; see Fig. 2 for the specificities of which label corresponded to which sample. The sample-label mixture was incubated for 1 h at room temperature. Labelling reaction was stopped by adding 8 μl of 5% hydroxylamine per sample.

**Fig. 1:**
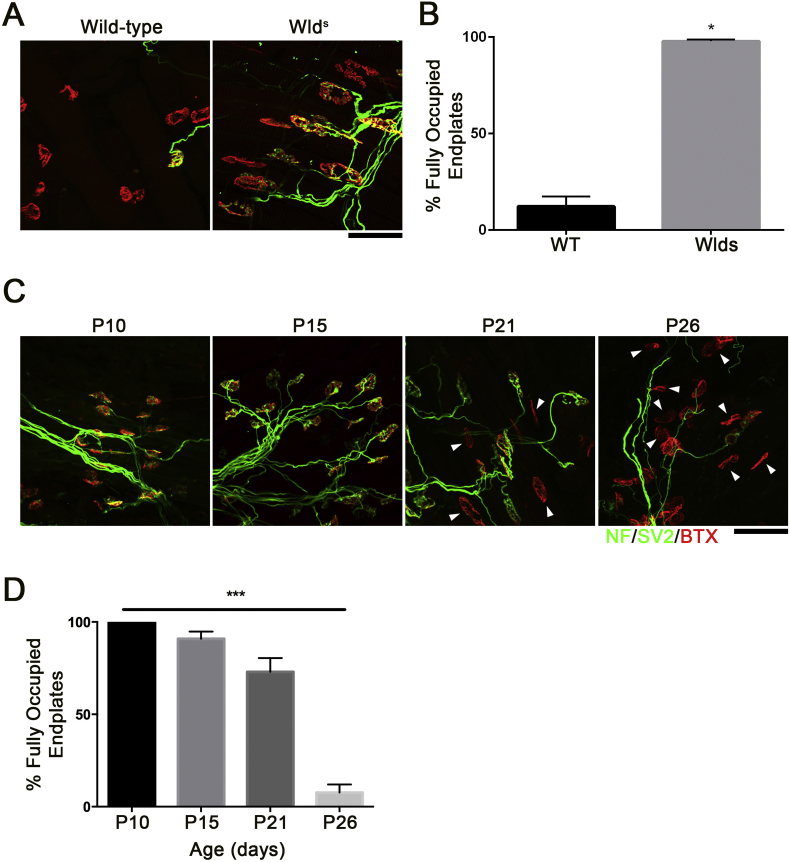
An *ex-vivo* system confirms decreased rate of axon degeneration in *Wld*^*s*^ and neonatal mice. (A) Representative confocal micrographs of NMJs from age-matched adult C57BL/6 J wildtype and *Wld*^*S*^ mouse lumbrical muscles after 24 h incubation *ex-vivo* at 28 °C in oxygenated ringer solution. Note the retention of NMJ integrity in the *Wld*^*S*^ model compared to the marked loss of pre-synaptic inputs in the wildtype muscle. Scale bar = 25 μm (B) Bar chart (Mean ± SEM) showing the percentage of fully occupied endplates 24 h post-axotomy reveals an increased in the percentage of fully occupied endplates in *Wld*^*S*^ mice compared to wildtype C57BL/6 J mice. (*p < 0.05 by Mann-Whitney *U* test; *n* = 3 preparations per group, data are Mean ± SEM). (C) Representative confocal micrographs of NMJs from the deep lumbrical muscles from P10, P15, P21 and P26 mice after 24 h incubation *ex-vivo* at 28 °C in oxygenated mammalian physiological saline. Note the increase in vacant endplates (denoted by arrowhead) at P21 and P26 compared to P12 and P15. Scale bar = 25 μm (D) Bar chart (Mean ± SEM) of the percentage of fully occupied endplates in the deep lumbrical muscles from P12, P15, P21 and P26 mice confirms an age-associated decrease in the percentage of fully occupied endplates. (***p < 0.001, by Kruskall-Wallis test; *n* = 3 preparations per group.

**Fig. 2:**
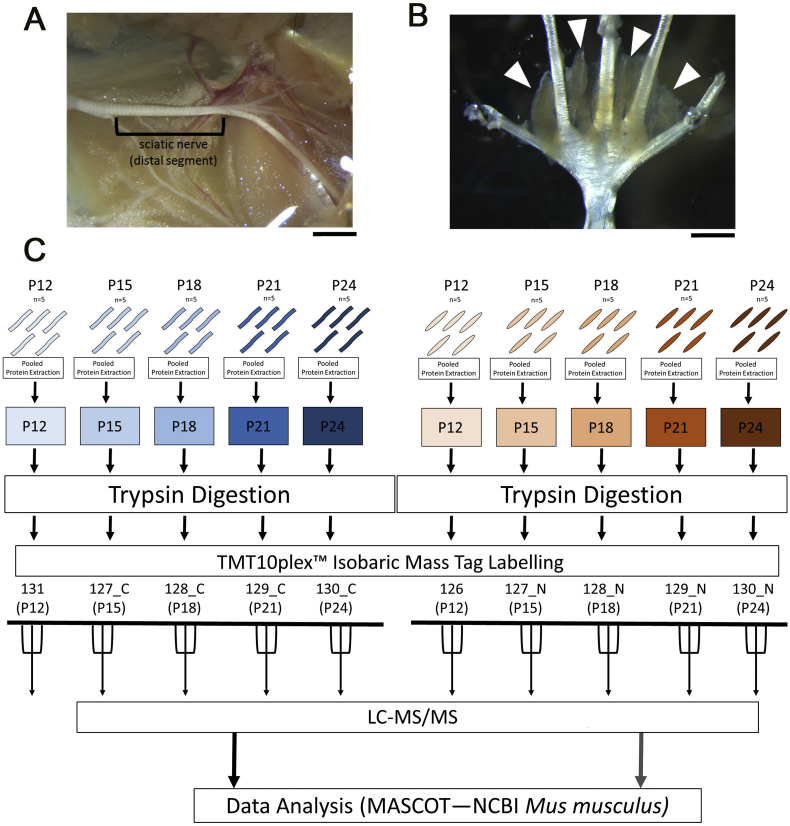
Schematic of sample preparation, TMT 10plex™ Tandem Mass Tag labelling, and quantitative LC-MS/MS workflow. (A, B) Images showing region of sciatic nerve (A) or lumbrical muscles (B) which were isolated for proteomics (C) Schematic of sample preparation workflow for TMT 10plex™ labelling and subsequent LC-MS/MS. Sciatic nerves (*n* = 5 mice, 10 nerves) and lumbrical muscles (5 mice, 20 muscles per timepoint) were pooled for a single extraction in label-free buffer per timepoint. Pooled samples were labelled using all 10 isobaric mass tags from a TMT 10plex™ kit (Thermo Fisher). Labelled samples were prepared for LC-MS/MS and run in technical triplicate. Scale bars = 2 mm.

Following labelling with TMT, samples were mixed, desalted, and dried in a speed-vac at 30 °C. Samples were re-dissolved in 200 μl ammonium formate (NH₄HCO₂) (10 mM, pH 10) and peptides were fractionated using an Ultimate 3000 RP-High pH High Performance Liquid Chromatography column (Thermo-Scientific) containing an XBridge C18 column (XBridge peptide BEH, 130 Å, 3.5 μm, 2.1 × 150 mm) (Waters, Ireland) with an XBridge guard column (XBridge, C18, 3.5 μm, 2.1X10mm) (Waters, Ireland). Buffers A and B used for fractionation consist, respectively, of (A) 10 mM ammonium formate in milliQ water and (B) 10 mM ammonium formate with 90% acetonitrile. Before use, both buffers were adjusted to pH 10 with ammonia. Fractions were collected using a WPS-3000FC auto-sampler (Thermo-Scientific) at 1 min intervals. Column and guard column were equilibrated with 2% Buffer B for twenty minutes at a constant flow rate of 0.2 ml/min. 175 μl per sample was loaded onto the column at a rate of 0.2 ml/min, and the separation gradient was started 1 min after sample was loaded onto the column. Peptides were eluted from the column with a gradient of 2% Buffer B to 5% Buffer B in 6 min, and then from 5% Buffer B to 60% Buffer B in 50 min. Column was washed for 16 min in 100% Buffer B and equilibrated at 2% Buffer B for 20 min as mentioned previously. The fraction collection started 1 min after injection and stopped after 80 min (total 80 fractions, 200 μl each). The total number of fractions concatenated was set to 15 and the content of the fractions was dried and suspended in 50 μl of 1% formic acid prior to analysis with LC-MS.

### 2.6 LC-MS/MS analysis

Liquid chromatography- tandem mass spectrometry was performed by FingerPrints Proteomics Facilities at the University of Dundee, to the following protocol: Analysis of peptide readout was performed on a Q Exactive™ HF Hybrid Quadrupole-Orbitrap™ Mass Spectrometer (Thermo Scientific) coupled with a Dionex Ultimate 3000 RS (Thermo Scientific). LC buffers were made up to the following: Buffer A (2% acetonitrile and 0.1% formic acid in Milli-Q water (*v*/v)) and Buffer B (80% acetonitrile and 0.08% formic acid in Milli-Q water (v/v). Aliquots of 15 μl per sample were loaded at a rate of 5 μl/min onto a trap column (100 μm × 2 cm, PepMap nanoViper C18 column, 5 μm, 100 Å, Thermo Scientific) which was equilibrated with 98% Buffer A. The trap column was washed for 6 min at the same flow rate and then the trap column was switched in-line with a resolving C18 column (Thermo Scientific) (75 μm × 50 cm, PepMap RSLC C18 column, 2 μm, 100 Å). Peptides were eluted from the column at a constant flow rate of 300 nl/min with a linear gradient from 95% Buffer A to 40% Buffer B in 122 min, and then to 98% Buffer B by 132 min. The resolving column was then washed with 95% Buffer B for 15 min and re-equilibrated in 98% Buffer A for 32 min. Q Exactive™ HF was used in data dependent mode. A scan cycle was comprised of a MS1 scan (*m*/*z* range from 335 to 1800, with a maximum ion injection time of 50 ms, a resolution of 120,000 and automatic gain control (AGC) value of 3 × 106) followed by 15 sequential-dependent MS2 scans (with an isolation window set to 0.4 Da, resolution at 60000, maximum ion injection time at 200 ms and AGC 1 × 105. To ensure mass accuracy, the mass spectrometer was calibrated on the first day that the runs were performed.

### 2.7 Database search and protein identifications

Raw MS data from the 15 fractions were searched against mouse (*Mus musculus*) protein sequences from UniProtKB/Swiss-Prot using the MASCOT search engine (Matrix Science, Version 2.2) through Proteome Discoverer™ software (Version 1.4, Thermo Fisher). Parameters for database search were as follows: MS1 Tolerance: 10 ppm; MS2 Tolerance: 0.06da; fixed modification: Carbamidomethyl (C) Variable Modification: Oxidation (M), Dioxidation (M), Acetyl (N-term), Gln- > pyro-Glu (N-term Q), TMT 10(N-term and K); maximum missed cleavage: 2; and target FDR 0.01.

All identifications were quantified as relative ratios of expression compared to the first time point (P12) through Proteome Discoverer™ software (Thermo Fisher, Version 1.4). Relative ratios along with UnitProtKB/Swiss-Prot identifications were exported into Microsoft Excel as a raw data file containing ID, ratio of change in expression at each time point (P15, P18, P21, P24) compared to P12 = 1.

### 2.8 Selection of relevant expression clusters from filtered data in BioLayout Express3D

Filtering raw proteomic data by an expression change of >20% at P24 than at P12 in produced a separate filtered list of proteins in nerve and muscle. These lists were imported separately into BioLayout Express3D (Theocharidis et al., 2009) and clustered based on relative expression profile from P12 to P24. Algorithms in BioLayout Express3D generate a visual network to represent each data set, utilizing special proximity to represent the similarity in expression profile of individual proteins over time. The resultant visual networks were utilized to distinguish expression clusters that followed either a general upward or downward trend in expression from P12 to P24. These “upregulated” and “downregulated” clusters in nerve and muscle respectively were analyzed on an individual basis to exclude clusters that did not follow an experimentally relevant expression profile from P12 to P24. For example, clusters containing proteins that exhibited a consistent increase or decrease in expression were selected, while clusters consisting of proteins that exhibited an overall upregulation or downregulation but oscillated in expression during middle time points were excluded.

### 2.9 DAVID analysis

Proteins exhibiting a consistent up-regulation or down-regulation in nerve or muscle from P12 through all four subsequent time points to P24, regardless of magnitude of change, were submitted as a gene list and converted into DAVID IDs against the DAVID 6.7 *Mus musculus* database(Huang et al., 2009). Four separate analyses were performed from the following lists of proteins, grouped as: (1) Consistent upregulated expression in nerve, (2) Consistent downregulated expression in nerve, (3) Consistent upregulated expression in muscle, and (4) Consistent downregulated expression in muscle. These lists were analyzed using the Functional Annotation Clustering tool in DAVID Bioinformatics Resources (Version 6.7) to produce a list of functional annotations. Functional annotations are ranked by a DAVID enrichment score. An enrichment score >1.3 in DAVID is equivalent to *p* < 0.05, and considered to be statistically significant.

### 2.10 In silico protein pathway analysis

The Ingenuity Pathway Analysis (IPA) application (Ingenuity Systems, Silicon Valley, CA) was used to visualize and explore the cellular and molecular pathways that may have been altered as a result of expression changes over time. IPA generates networks of gene and protein interactions and disease associations, amongst other results, based on *in silico* predicted or experimentally reported interactions stored within the “hand-curated” Ingenuity Knowledge database(Wishart et al., 2012; Savli et al., 2008). The majority (90%) of data comprising the Ingenuity Knowledge database are derived from peer-reviewed publications; the remaining 10% of stored interactions have been identified by other *in silico* techniques. Networks generated using IPA were set to a maximum of 35 member molecules, and were ranked according to a score calculated by a right-tailed Fisher's exact test, which considers total protein input and size of the produced network. Network scores represent the relevance of the particular network to the overall analysis (*i.e.* proteomic alterations in nerve or muscle between P12 and P24). In this study, networks generated using IPA were limited to those producing the top 25 scores. For more information on the computational methodology underpinning IPA, please refer to http://www.ingenuity.com/.

Separate pathway analyses were performed on the filtered nerve and muscle data sets, which produced 25 predictive molecular networks each. A third, combined analysis, which consolidated nerve and muscle results, enabled the identification of molecular overlap within networks generated from nerve and muscle data. Extrapolation of this overlap through the Pathway Designer function generated a combined network in which molecular interactions conserved between both nerve and muscle data sets were explored.

### 2.11 Complex I activity assay

Mitochondrial OXPHOS Complex I activity was determined by immunocapture ELISA kit following the manufacturer's instructions (ab109721). Nerve (sciatic, brachial plexus, intercostal nerves) and muscle (deep lumbrical muscle) were isolated from recently sacrificed mice and snap-frozen on dry ice prior to assay. Thawed tissue was homogenized in ice-cold PBS with Dounce homogenizer and protein extracted with detergent solution at a concentration of 1:10. 250 μg of protein was incubated to the wells of microplate pre-coated with Complex I capture antibody for 3 h at room temperature. Activity of immunocaptured Complex I enzyme (NADH dehydrogenase) was determined by measuring the oxidation of NADH to NAD+ and simultaneous reduction of a dye which leads to increased absorbance at 450 nm. Each reaction was performed in duplicate. Activity was expressed as a change in absorbance per minute per quantity of protein per reaction, and values are expressed normalized to P12.

### 2.12 Quantitative fluorescent western blotting

Levels of Complex I protein were determined by quantitative fluorescent western blot (QFWB). Gastrocnemius muscles were isolated from recently sacrificed mice and snap-frozen on dry ice prior to immunoblotting. Thawed tissue was homogenized in ice-cold PBS with Dounce homogenizer and extracted in RIPA buffer (Fisher Scientific) with 1% Halt protease inhibitor cocktail (Sigma Aldrich). Concentration of protein was determined by micro-BCA assay (Pierce Biosystems). Samples were prepared to load 25 μg of protein in 10 μl deionised water and 5 μl of NuPage® LDS Sample buffer 4× (Invitrogen, UK) and run on a NuPAGE™ Novex™ 4–12%Bis-Tris protein gel (Invitrogen, UK) before transfer to a PVDF membrane using the i-Blot2® transfer system (Invitrogen, UK).

To determine total protein concentration for a loading control, membranes were incubated in 5 ml of Ponceau S solution; 0.1% Ponceau S (Sigma-Aldrich), 0.5% acetic acid (Sigma-Aldrich) in ddH2O at room temperature for 30 min before washing in ddH2O until bands were visible. Total protein image was scanned in greyscale using a CanoScan LiDE220 digital scanner (Canon) and saved as a TIFF image for total protein analysis.

For QFWB, membranes were blocked in 5 ml Odyssey® Blocking Buffer (Li-COR Biosciences) at room temperature for 30 min and incubated in primary antibody solution containing primary antibodies at the following concentration (SIRT2 1:500, Abcam; Igf2 1:1000, Abcam; Total OXPHOS 1:1000, Abcam) with 1% tween-20 (Sigma) in 5 ml Odyssey® Blocking Buffer (Li-COR Biosystems) at 4 °C overnight. Antibody to SIRT2 was generously provided by members of the Gillingwater laboratory. Membranes were incubated in secondary antibody solution containing either IRDye® 680RD donkey anti-mouse IgG (H + L) or IRDye® 680RD donkey antirabbit IgG (H + L) antibodies (Li-COR Biosciences) at a concentration of 1:5000 (0.02%) in a solution of 1% tween-20 (Sigma) in 5 ml Odyssey® Blocking Buffer (Li-.

COR Biosciences for 90 min at room temperature before drying and storage at 4 °C prior to imaging and analysis. For measurement and analysis, TIFF images of Ponceau-stained blots were imported into Odyssey® ImageStudio Lite software (Version 5.2). ImageStudio Lite (Version 5.2) was used to analyze the intensity of identical sections of total protein banding against background. Readouts of the intensity of total protein banding relative to background were imported into Microsoft Excel (Windows 2013) to calculate loading consistency and normalization factors. Western blots were imaged on the 700 nm channel using the Odyssey® Infrared Imaging System at a resolution of 169 μm. All quantification was performed on the 700 nm channel, with the intensity of bands normalized using the factors generated from total protein analysis. All statistical analysis and generation of graphs was performed in GraphPad Prism7 (Windows). Individual statistical tests used are noted in figure legends. Statistical significance was considered to be *p* ≤ 0.05.

### 2.13 Dorsal root ganglion cultures

Briefly, E16 rat embryos were decapitated, and the limbs and organs were removed. The spinal cord with dorsal root ganglia (DRGs) was dissected and placed in a Petri dish containing cold L-15 medium (Gibco, 11,415–064). For DRG explants, complete DRGs were cultured in 24-well dishes containing 400 μl of Neurobasal medium, 2% B27, 0.3% l-glutamine, 1% streptomycin/penicillin, 4 μM aphidicolin (Sigma, A0781), 7.5 μg/ml 5-fluoro-2- deoxyuridine (Sigma, F0503), and 50 ng/ml nerve growth factor (NGF) (Invitrogen, 13,257–019). The mixture of aphidicolin and fluoro-2-deoxyuridine inhibits proliferation of Schwann cells by inhibition of DNA polymerase (Spadari et al., 1985; Wallace and Johnson Jr., 1989), thus constituting a highly pure sensory neuron culture (Heermann et al., 2012). DRGs were cultured for 7–11 days at 37 °C and 5% CO_2_. Axotomy of DRG explants was made using a micropipette tip to separate all the axons from their somas.

### 2.14 Axonal degeneration index

Number of axons per area of nerve tissue was assessed in confocal images of neurofilament-immunostained explant sections (matched for laser power, photomultiplier tube gain/offset, and post processing) using the particle analysis macro of ImageJ. Relative neurite integrity was based on the ratio of the areas of fragmented axons *versus* total axonal area (Villegas et al., 2014). Degenerated axon fragments were detected using the particle analyzer algorithm of ImageJ (NIH, USA) and the total fragmented axon area *versus* total axonal area was used to estimate a degeneration index.

### 2.15 DHE assay

Superoxide levels were measured using dihydroethidium (DHE) fluorescence 6 h after DRG axotomy in an epifluorescence microscope. After axotomy, neurites were incubated in the last 30 min with 5 M DHE under culture conditions. DHE fluorescence was obtained under a Cy5 emission filter. Superoxide levels on neurites were determined using NIH ImageJ colocalization highlighter plugin. Oxidative stress levels were calculated as the ratio of DHE fluorescent signal area in neurites. Briefly, DHE signal and phase-contrast colocalization were used to obtain DHE area and neurite length. Photoshop (Adobe Systems) and NIH ImageJ were used to perform this analysis.

## 3 Results

Previous work has demonstrated that axotomised NMJs from neonatal mice showed a reduced rate of WD compared to NMJs from adult mice under otherwise identical experimental conditions (Murray et al., 2011). Between postnatal day 12 (P12) and P24 the rate of axotomy induced degeneration accelerates until the adult response of rapid synaptic degeneration is established. We first sought to determine whether age-dependent regulation of synaptic degeneration could be replicated using an “*ex-vivo”* assay of synaptic degeneration (Brown et al., 2015). Nerve-muscle preparations, comprising the tibial nerve and all branches innervating the attached deep lumbrical muscles were swiftly isolated from euthanized mice, and maintained in oxygenated physiological solution, then assayed for innervation 24 h later. This protocol mimics axotomy *in vivo* (Brown et al., 2015). Comparison of endplate occupancy at immunostained NMJs from the deep lumbrical muscles from adult *Wld*^*S*^ mice revealed that, as expected, the percentage of fully occupied NMJs was significantly greater than that of NMJs from age-matched wild-type C57BL/6 adults (Fig. 1A, B). Thus, this assay is sufficiently sensitive to reveal factors that influence the rate of WD. Equivalent preparations were isolated from mice aged P10 to P26. Analysis of NMJs from the lumbrical muscles revealed a significant decrease in the percentage of fully occupied endplates between P10 and P26 (Fig. 1C,D). Together, the data suggest that an *ex-vivo* model of traumatic injury can be used to detect differences in the rate of axon degeneration and confirms the age-associated decrease in the rate of axon degeneration in mice aged between P10 and P26.

### 3.1 Tandem mass tagging (TMT) quantitative proteomics produces a robust and comprehensive coverage of the nerve and muscle proteome

The combination of published work and the data presented above clearly demonstrate a marked increase in the rate of WD during the neonatal time period. We therefore reasoned that whatever regulates this progressive increase in the rate of WD, may also be changing progressively over the time period between P12 and P24. Given that the process of WD occurs after the axonal and synaptic compartments of the cell are severed from the cell body, and the overall speed of WD, we reasoned that the molecular regulators of this process would be most likely evidenced at the protein level, as opposed to at the level of transcription. We therefore performed a comprehensive proteomic analysis of muscle and nerve between P12 and P24, with the goal of identifying proteins and cellular processes which demonstrated a steady alteration correlating with the change in the rate of axon degeneration.

For this analysis, we employed tandem mass tagging (TMT) based proteomics using a 10plex (TM) labelling kit to allow comparative protein profiling across ten different sample groups. For this analysis we collected deep lumbrical muscle and sciatic nerve samples from mice aged P12, P15, P18, P21 and P24 and performed TMT proteomic analysis (Fig. 2). Consolidation of raw peptide outputs across all five time points within sciatic nerve and muscle samples respectively were translated into protein identifications by searching against murine protein sequences using the MASCOT protein database (Matrix Science, Version 2.2) and quantified expression as relative ratios compared to the first time point (P12) in each tissue type. This produced a list of protein identifications and ratios of expression/detection throughout the time course for muscle and nerve (see Methods for more information). Both nerve and muscle analyses are traditionally faced with the problem of detection saturation due to particularly abundant protein groups *i.e.* myelin related proteins in nerve or myosin groups in muscle (Comley et al., 2011; Mutsaers et al., 2013, 2011). The application of TMT combined with developments in sample handling workflows (smaller sample requirements, reduced processing losses, greater fractionation control) and recent technological advances in mass spectrometry sensitivity, has resulted in an unexpectedly high number of reliable protein identifications in all sample groups.

Within the sciatic nerve, 7440 total proteins were identified, of which 5625 were identified by 2 or more unique peptides (Supplementary Table 1). Within the lumbrical muscles, 6079 total proteins were identified, of which 4414 were identifiable by >2 unique peptides (Supplementary Table 2).

Having identified so many proteins in both nerve and muscle, we applied a filtering protocol in order to identify protein alterations which are likely to be biologically relevant to the phenomenon of early age dependent neuronal stability. Thus, we began by further filtering the proteomic output to exclude any proteins with altered expression <20% in either direction at P24 compared to P12. Previous studies in neural tissue and skeletal muscle have demonstrated that the 20% cut-off threshold produces a reliable list of candidate molecules (Comley et al., 2011; Mutsaers et al., 2013, 2011; Aghamaleky Sarvestany et al., 2014; Fuller et al., 2014).

**Fig. 3:**
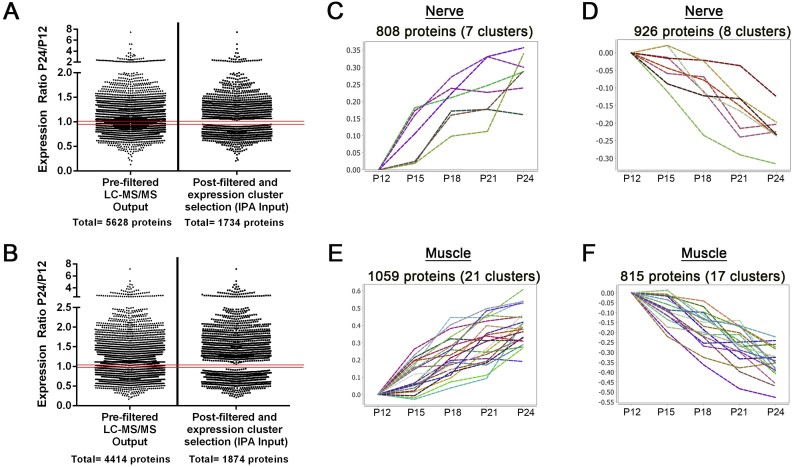
Filtering of raw proteomic data produces an analysis-ready dataset of experimentally relevant proteins. (A,B) Scatter plot depicting the filtering process undertaken on raw proteomics nerve data (LC-MS/MS output identified by 2 more unique peptides) through manual selection of relevant expression clusters to generate a final list of 1734 proteins exhibiting consistent increases or decreases in expression in the murine sciatic nerve (A) or lumbrical muscle(B). Data are presented as expression ratios (P12/P24) and each data points represents an individual protein. (C-F) Graphs displays the relative change in protein levels relative to P12 of protein clusters, clustered on the basis of a similar trend, which display a desirable profile of continuous increase (C,E) or continuous decrease (D,F) in levels in either sciatic nerve (C,D) or lumbrical muscle (E,F). Cluster generated in BioLayout *Express*^*3D*^ from the filtered nerve dataset (exhibiting a > 20% change in relative expression at P24 from P12). Proteins within these clusters feature a steady relative increase or decrease in levels over the P12-P24 time period, and were therefore selected for future analysis.

In order to identify proteins whose expression are likely to correlate with the differential vulnerability described above, we used the network-based software BioLayout *Express*^*3D*^. This software uses complex pattern recognition algorithms to cluster data by user-defined dimensions, while generating visual networks that utilize spatial proximity to represent relatedness. By clustering our refined protein list based on protein expression profile over the 5 time points, it was possible to visualize and isolate clusters of proteins exhibiting similar expression profiles over the time-course. We were therefore able to further filter the nerve and muscle data sets to include only those candidates which exhibited a desirable expression profile over the experimental P12-P24 period (Fig. 3). Specifically, clusters which exhibited a relatively consistent up-regulation or down-regulation in expression from P12 to P24 were selected for further analysis. Conversely, clusters containing proteins which showed a discontinuous expression profile, for example, those oscillating between an increase and decrease over the experimental period, were excluded. Through this filtering process, we produced a consolidated data set containing only proteins exhibiting a relative change in expression >20% at P24 relative to P12 and an experimentally relevant expression profile (Supplementary Tables 3 and 4).

**Fig. 4:**
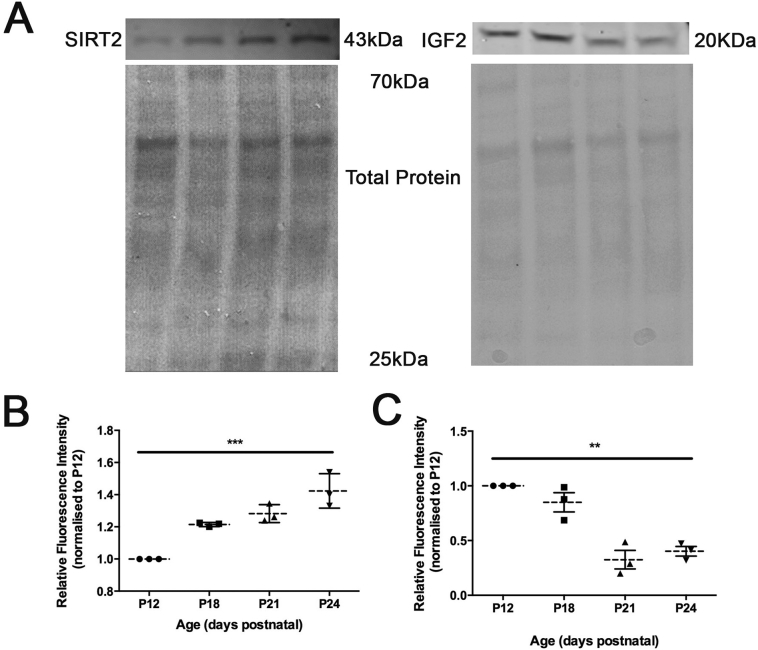
Candidate validation of proteomic data by quantitative fluorescent Western blotting. (A) Representative western blots for NAD-dependent deacetylase sirtuin-2 (Sirt2) in nerve and insulin-like growth factor 2 (Igf2) in muscle. All candidate blots were normalized to total protein load (Ponceau S stain) (B,C) Scatter plots (individual data points; Mean ± SEM) of relative expression of Sirt2 (B) and Igf2 (C) relative to P12. Note direction and magnitude of change is consistent with TMT data. (n = 3 pairs of nerves (SIRT2) or 8 lumbrical muscles (IGF2) per time point; **p < 0.01, ***P < 0.001 by Kruskall-Wallis test).

To validate the proteomic data, we performed quantitative fluorescent western blotting on protein candidates selected from the raw LC-MS/MS output. Candidates were selected on the basis of their magnitude of change over the experimental period, implication in injury-induced or disease-related neuronal degeneration, and availability of quality antibodies suitable for fluorescent-based western blotting. For example, the NAD-dependent deacetylase sirtuin-2 (SIRT2) exhibited an increase in the proteomic data (1.330 P24/P12 ratio in nerve) and has been demonstrated to be up-regulated across a number of neurodegenerative disease and injury models (Allodi et al., 2016; Amorim et al., 2015; Graham et al., 2017; Hedlund et al., 2010; Murray et al., 2015; Zhang et al., 2013). Quantitative florescent western blot analysis confirmed a consistent increase in protein levels observed between P12 and P24 (1.44 ± 0.06, P24/P12 ratio of Mean ± SEM; Fig. 4A,B). Additionally, the neurotrophic factor insulin-like growth factor 2 (IGF2) exhibited a decrease in the proteomic data (0.410 P24/P12 ratio in muscle) and has been implicated in in a mouse models of motor neuron disease (Allodi et al., 2016; Hedlund et al., 2010; Murray et al., 2015; Zhang et al., 2013). Quantitative fluorescent western blot analysis on protein extracted from lumbrical muscle (IGF2) confirmed a consistent decrease in protein levels observed between P12 and P24 (0.402 ± 0.044, P24/P12 ratio of Mean ± SEM; Fig. 4A,C). Thus, these results confirm the direction and magnitude of expression change reported in our proteomic profiling.

### 3.2 Functional annotation analysis of filtered nerve and muscle data reveals a conserved enrichment for proteins related to oxidative phosphorylation and mitochondrial bioenergetics

The refined nerve and muscle data sets were sorted into four lists: (1) consistent up-regulated expression in nerve, (2) consistent down-regulated expression in nerve, (3) consistent up-regulated expression in muscle and (4) consistent down-regulated expression in muscle. These lists were analyzed using the Functional Annotation Clustering tool in DAVID Bioinformatics Resources (Version 6.7) to determine if the proteins whose expressions trend together share functional annotations. Interestingly, consistently up-regulated protein groups within the sciatic nerve revealed a marked enrichment for processes implicated in oxidative phosphorylation and mitochondrial respiration (Table 1). The most significantly enriched group was comprised of proteins involved in NAD-dependent “redox” processes. Analysis of proteins consistently down-regulated within the sciatic nerve was dominated by the presence of biological groups involved in mRNA processing and binding, as well as RNA-editing machinery such as the RNP complex and spliceosome (Table 1). Other top groups contained proteins pertaining to endoplasmic reticulum activity.

**Table 1:** Top 5 most significantly enriched functional clusters in sciatic nerve.

Up from P12-P24		Down from P12-P24	
Cluster name	Enrichment score	Cluster name	Enrichment score
Mitochondrial oxidation reduction; NAD-dependent	8.44	mRNA processing/Spliceosome	25.24
Cell fraction	4.61	RNP complex/ribosome	23.19
Cytoskeletal actin binding	4.53	RNA binding	16.1
Membrane lipoproteins	4.26	Intracellular organelle lumen	12.54
Magnesium binding	4.06	Endoplasmic reticulum	11.87

In the muscle, analysis of proteins consistently up-regulated also revealed an enrichment of processes relating to mitochondria, specifically (as with nerve) oxidative phosphorylation (Table 2). Indeed, all “top-5” biological groups produced by this analysis were clusters involved in electron transport chain activity or broader mitochondrial functions. Analysis of proteins consistently down-regulated within the lumbrical muscles identified endoplasmic reticulum (similar to nerve), protein transport, membrane-bound vesicles, and actin-binding (Table 2). Thus, the functional clustering analysis suggest a general shared consistent increase in proteins pertaining to mitochondria, oxidation reduction and the electron transport chain in muscle and nerve.

**Table 2:** Top 5 most significantly enriched functional clusters between P12 and P24 in the lumbrical muscles.

Up from P12-P24		Down from P12-P24	
Cluster name	Enrichment score	Cluster name	Enrichment score
Mitochondria	134.15	Endoplasmic reticulum	12.26
Mitochondrial membrane	82.63	Protein transport	10.38
Oxidation reduction/electron transport chain (ETC)	43.65	Membrane-bound vesicle	9.81
Electron transport/oxidoreductase/NADH dehydrogenase activity	25.4	Actin binding/actin cytoskeleton	8.89
Cellular respiration/electron transport chain	22.5	Actin cytoskeleton organization	8.52

### 3.3 *In silico* pathway analysis reveals conserved direct interactions between components of the oxidative phosphorylation pathway in both muscle and nerve

**Supplementary Fig. 1:**
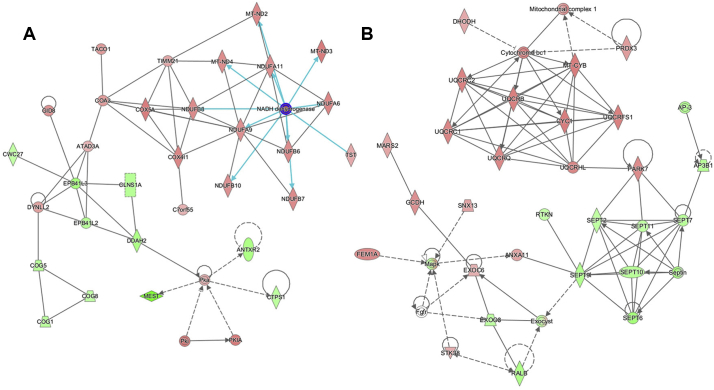
IPA analysis in muscle reveals pathways pertaining to the inner mitochondrial membrane and oxidative phosphorylation. A, B) Interaction networks generated using IPA software showing proteins which were consistently down (green) or up (red) regulated in lumbrical muscle between P12 and P24 arranged by known interactions. Intensity of colour reflects magnitude of change. White represents a molecule that is absent from the datasets but an important component of the network. Solid connecting lines represent a direct interaction, while dashed connecting lines indicate an indirect interaction. Blue represents highlighted direct interactions of NADH dehydrogenase (Complex I). Note enrichment of proteins pertaining to Complex I and IV of the electron transport chain.

**Supplementary Fig. 2:**
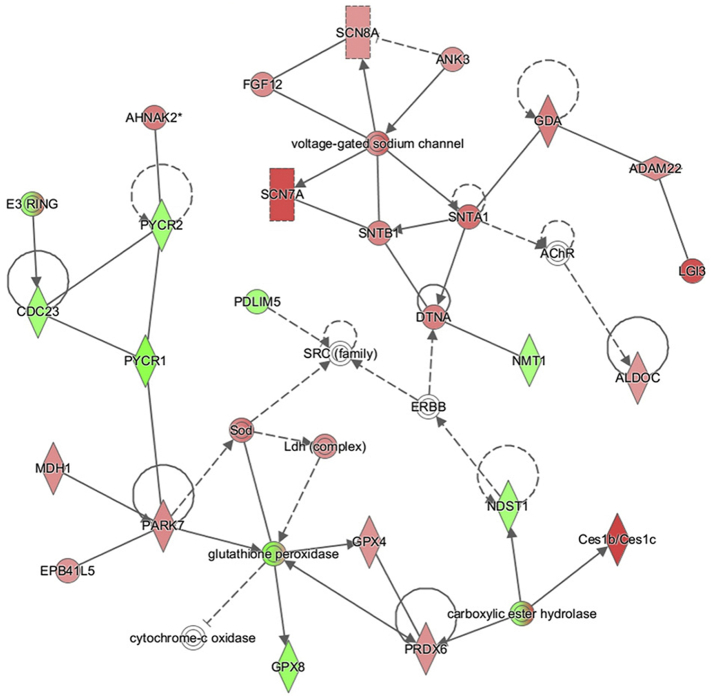
IPA analysis in nerve reveals pathways pertaining ion channel localization and protective response to oxidative stress. Interaction networks generated using IPA software showing proteins which were consistently down (green) or up (red) regulated in sciatic nerve between P12 and P24 arranged by known interactions. Intensity of colour reflects magnitude of change. White represents a molecule that is absent from the datasets but an important component of the network. Solid connecting lines represent a direct interaction, while dashed connecting lines indicate an indirect interaction. Note enrichment of proteins pertaining to ion channel localization and the protective response to oxidative stress.

Given the enrichment for proteins associated with mitochondrial functions within our refined candidate lists, we next employed IPA software to our cluster-derived lists in order to contextualize these protein changes into specific cellular cascades with the aim of identifying potential intervention points, as performed previously (Catenaccio et al., 2017; Graham et al., 2017; Llavero Hurtado et al., 2017). Unlike other network-based approaches, IPA generates molecular networks based on interactions reported in its “hand-curated” database, with the option for restricting analyses to only experimentally reported interactions. Initially, this analysis was performed on nerve and muscle data sets independently. This analysis of nerve and muscle included all mappable proteins from both nerve and muscle data sets (1713 and 1862 respectively) and were analyzed in parallel, with interactions restricted to only those reported experimentally (as opposed to *in silico* predictions). The filtered list of proteins from nerve and muscle were imported into IPA and of these, >98% were mappable by the software and therefore suitable for data mining of the published literature to generate interaction networks. As expected, this revealed molecular networks dominated by proteins pertaining to mitochondrial respiratory components, specifically, components of the electron transport chain—and redox sensitivity in both muscle and nerve (Supplementary Fig. 1, Supplementary Fig. 2).

**Fig. 5:**
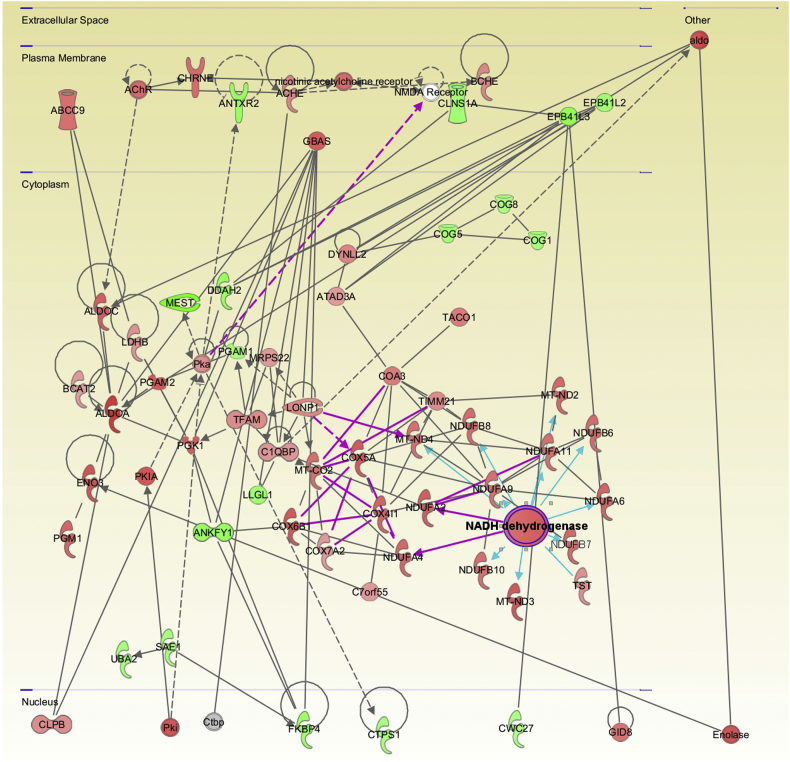
Combined analysis in nerve and muscle reveals conserved escalation in mitochondrial oxidative phosphorylation related proteins. Use of the Path Designer function in IPA software, to reveal combined interaction network, reveals an overlap in molecular interactions pertaining to the mitochondrial oxidative phosphorylation process. Specifically, candidates identified as components of mitochondrial Complex I (NADH dehydrogenase) and Complex IV (cytochrome *c* oxidase) exhibit conserved expression changes in both nerve and muscle data sets. Proteins within this network reflect those which up-regulated (red) and down-regulated (green) during the P12-P24 time-course in either nerve or muscle; intensity of colour corresponds with magnitude of expression change. White represents a molecule that is absent from both datasets but an important component of the network. Grey represents a molecule that is present in the datasets but the change in levels is below the 20% cut-off. Solid connecting lines represent a direct interaction, while dashed connecting lines indicate an indirect interaction. Interactions conserved between nerve and muscle data sets are highlighted as purple connecting lines. Blue connecting lines represent the highlighted interactions of NADH dehydrogenase. All suggested indirect interactions were confirmed manually using the IPA software to identify publications indicating an experimentally reported interaction between the two components. (For interpretation of the references to colour in this figure legend, the reader is referred to the web version of this article.)

We next utilized the Path Designer function in IPA to identify a molecular overlap between the predicted networks generated from the previous independent nerve and muscle analyses. Extrapolation of this convergence into a visual network, organized by cellular location, revealed the conservation of multiple direct interactions between components of the electron transport chain in both nerve and muscle data sets, with a specific enrichment for factors involved with Complex I and IV (Fig. 5). In accordance with the previous functional annotation analyses of nerve and muscle data sets, these results demonstrated an increased expression in proteins involved in the mitochondrial electron transport chain over the P12-P24 period. This suggests that differences in mitochondrial electron transport chain component expression and/or activity may serve as a key hub for the regulation of the molecular processes being mapped out through this temporal profiling of progressive vulnerability.

**Fig. 6:**
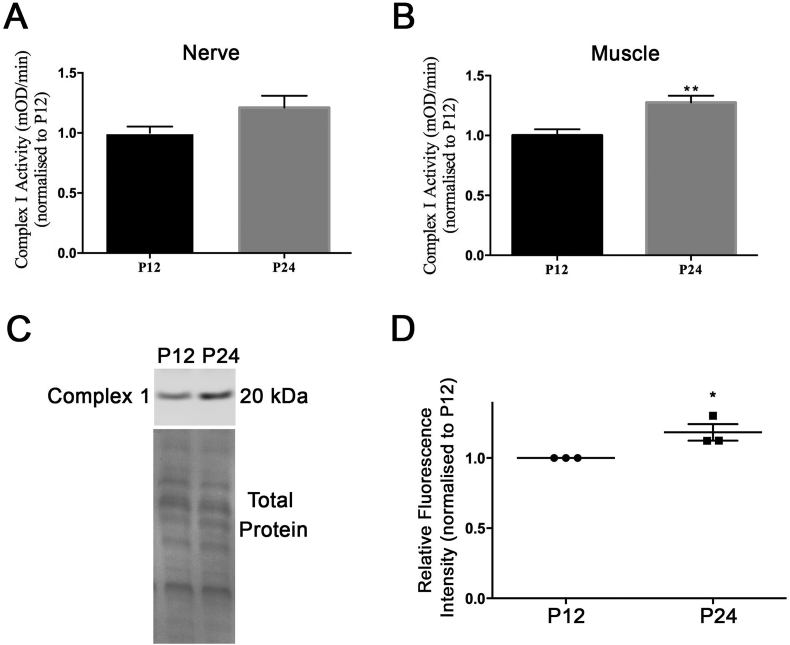
Complex I activity and levels are up-regulated between P12 and P24 in muscle and nerve. *(*A, B) Bar chart (Mean ± SEM) showing Complex I (NADH dehydrogenase) activity in P12 and P24 sciatic nerve (A) and lumbrical muscle (B). NADH dehydrogenase activity was measured kinetically over 60 min and the rate was determined as change in optical density over time, and expressed normalized to P12 (n = 3 biological replicates, 5 mice per group; ** *P* < 0.05 by Mann Whitney *U* test). (C) Image showing example western blot using antibodies against Complex I subunit on muscle from P12 and P24 mice, with Ponceau S staining showing total protein as a loading control. (D) Bar chart (Mean ± SEM) showing quantification of western blot against Complex I. Data expressed normalized to loading control and relative to levels at P12. (*N* = 3 biological replicates, 5 mice per time point, * P < 0.05 by Mann Whitney U test).

We next performed enzyme-linked immunosorbance assays (ELISA) of Complex I NADH dehydrogenase activity in order to determine whether our *in silico-*derived suggestion of an increase in Complex I expression was accompanied by a functional increase of Complex I activity*.* Analysis of these results suggested a consistent increase of Complex I activity in both the nerve and skeletal muscle of P24 compared to P12 mice, which reached statistical significance in muscle (Fig. 6A,B). We also aimed to assess the expression of Complex I by quantitative fluorescent western blotting, and confirmed an increase in Complex I expression levels in the skeletal muscle of P24 mice compared to P12 mice as predicted (Fig. 6C,D). This therefore confirms the validity of the data filtering strategy employed and the identified molecular cascades being represented in the subsequent *in silico* analysis.

### 3.4 Inhibition of Complex I prevents the rise in reactive oxygen species and protects axons following injury

**Fig. 7:**
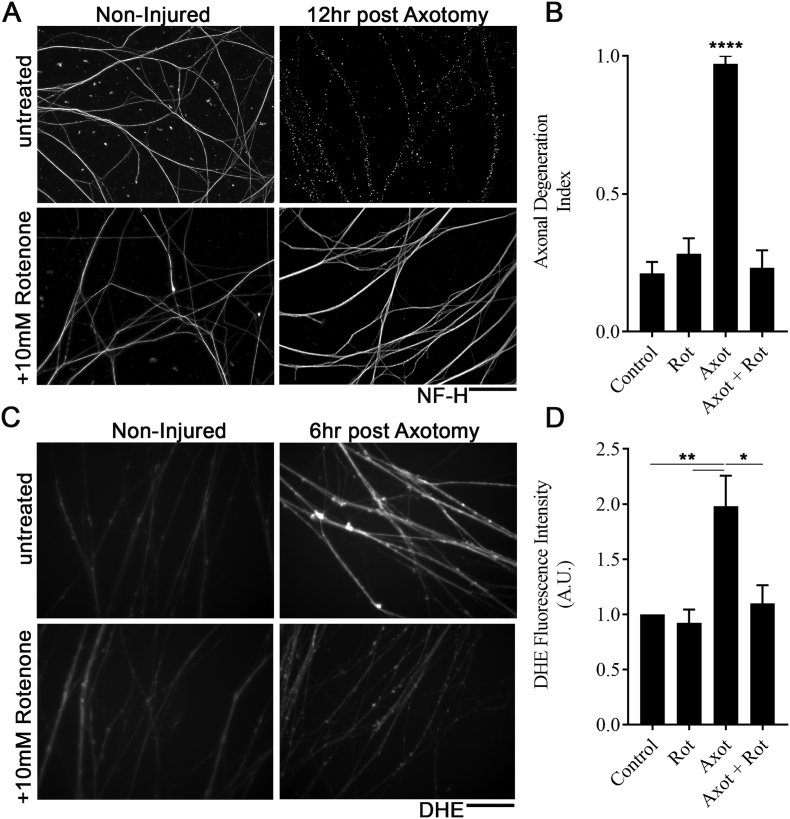
Rotenone protects axons and ameliorates rise in ROS in a DRG model of axon injury. A) Fluorescent images show axons from dorsal root ganglion primary cultures labelled with antibodies against neurofilament heavy chain (NF-H) which are either non-injured or 12 h post axotomy. Note widespread axon fragmentation following axotomy which is prevented by treatment with 10 μM rotenone. Scale bar = 20 μM B) Bar chart (Mean ± SEM) shown axon degeneration index in non-injured (Control/Rot) on injured (Axot/Axot + Rot) cultures which were either untreated (Control/Axot) or exposed to 10 μM Rotenone (Rot/Axot+Rot). **** *P* < 0.0001 Axot compared to all other groups, ANOVA with Tukeys multiple comparison test. C) Fluorescent images show axons from dorsal root ganglion primary cultures labelled with DHE which are either non-injured or 6 h post axotomy. Note increase in fluorescence following axotomy which is not observed following treatment with 10 μM rotenone. Scale bar = 20 μM D Bar chart (Mean ± SEM) shown DHE fluorescence in non-injured (Control/Rot) on injured (Axot/Axot + Rot) cultures which were either untreated (Control/Axot) or exposed to 10 μM Rotenone (Rot/Axot + Rot). **P < 0.01; *P < 0.05; ANOVA with Tukeys multiple comparison test.

The above data indicate that an increase in Complex I of the mitochondrial respiratory chain correlates with the acceleration in the rate of axon degeneration in response to injury. We therefore aimed to determine whether inhibition of Complex I could delay axon degeneration. For these experiments, we employed the Complex I inhibitor, rotenone and used an established murine dorsal root ganglion primary culture model (Lopez-Leal et al., 2018). This model has the benefit of allowing us to assess axon intrinsic factors regulating axon degeneration. 12 h after axotomy (induced by severing cell bodies from their axons), robust degeneration was observed in control cultures, as evidence by widespread fragmentation of neurofilament labelled axons (Fig. 7A). However, in cultures exposed to 10 μM rotenone, axonal fragmentation was visibly reduced. Quantitation of the degree of axon degeneration revealed a significant decrease in cultures treated with rotenone, to levels similar to those of non-injured controls (Fig. 7B), demonstrating that inhibition of Complex I can indeed be protective to the axonal compartment of neurons.

Reactive oxygen species (ROS) have previously been implicated in axon degeneration (Barrientos et al., 2011; Villegas et al., 2014; O'Donnell et al., 2013; Park et al., 2013; Calixto et al., 2012). As Complex I is a major source of reactive ROS, we hypothesized that the protection following Complex I inhibition may be linked to a decrease in ROS. Dihydroethidium (DHE; a Redox sensitive fluorescent probe) was applied to DRG cultures. 6 h following axon injury, a significant increase in DHE fluorescence was observed compared to non-injured controls (Fig. 7C,D). However, in cultures treated with rotenone, DHE fluorescence remained similar to non-injured controls, and was significantly reduced compared to untreated cultures. Together, these data suggest that inhibition of Complex I *via* rotenone can inhibit the axotomy related rise in ROS and prevent axon degeneration.

### 3.5 Up-regulating basal OXPHOS at P15 accelerates degeneration of NMJs

**Fig. 8:**
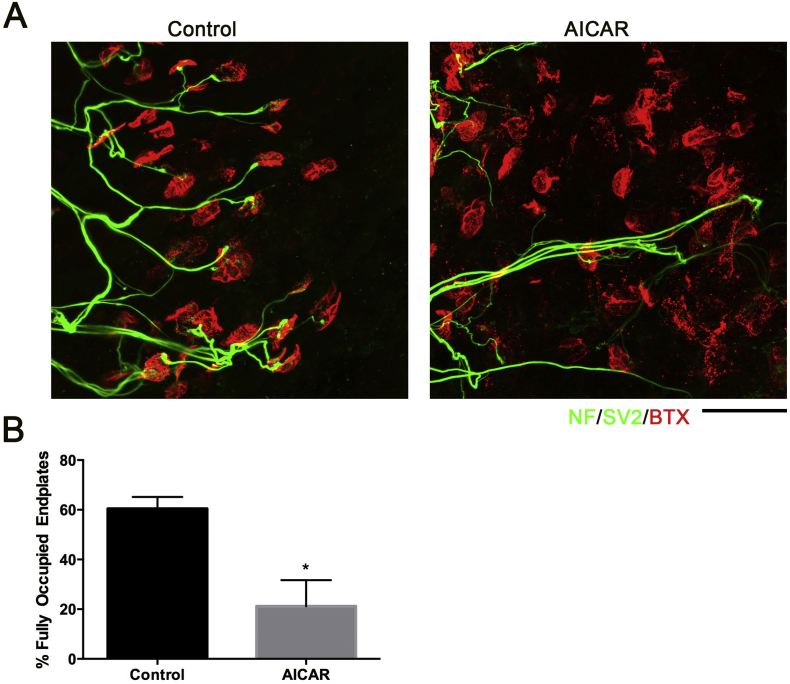
The AMP-kinase agonist AICAR accelerates NMJ loss in P15 mice in an *ex-vivo* model of axonal injury. (A) Representative confocal micrographs of NMJs from deep lumbrical muscles isolated from P15 C57BL/6 J wildtype mice, after 24 h incubation *ex-vivo* at 28 °C in either standard MPS CO_3_ ringer solution or MPS CO_3_ ringer solution +2 mM AICAR. NMJs labelled with antibodies against neurofilament (NF; green) and synaptic vesicle protein 2 (SV2; green) and alpha-bungarotoxin (BTX; red). Note increase in the number of vacant endplates following AICAR treatment. Scale bar = 30 μm (B) Bar chart (Mean ± SEM) confirms significant decrease in the percentage of fully occupied NMJs in AICAR-incubated muscles compared to control. (*n* = 4 muscles/4 mice per group; *p < 0.05 by Mann-Whitney *U* test). (For interpretation of the references to colour in this figure legend, the reader is referred to the web version of this article.)

The data thus far reveal an up-regulation in oxidative phosphorylation between P12 and P24, which coincides with the acceleration of WD with postnatal age. This data support the hypothesis that a decrease in Complex I activity results in the neuroprotective phenotype observed in neonatal mice. We therefore asked whether experimentally upregulating basal OXPHOS levels would abolish the delayed WD phenotype in neonatal mice. Adenosine monophosphate kinase (AMPK) is capable of sensing AMP levels and impacts upon downstream pathways, most markedly those acting upon the energy status of the cell. Activation of AMPK *via* the AMPK agonist AICAR results in up-regulation of oxidative metabolism (Golubitzky et al., 2011; Jager et al., 2007; Pacelli et al., 2015). In order to determine whether stimulation of mitochondrial activity could accelerate axon degeneration, we used an *ex-vivo* assay of nerve-muscle preparations from P15 mice in either the presence of absence of AICAR. Addition of 2 mM AICAR significantly enhanced the rate of NMJ degeneration after 24 h *ex-vivo* (Fig. 8). This suggest that increasing the amount of proteins related to (and thus overall activity of) oxidative phosphorylation between P12 and P24, could account for the acceleration of WD *in vivo*. Together with the findings above, these data support the notion that the changes in mitochondrial proteins observed between P12 and P24 could account for the acceleration in the rate of WD in this same time window.

## 4 Discussion

In this study we applied a tandem mass tagging method to quantitatively profile the proteome of the nerve and muscle over a critical period of postnatal development when the rate of WD following axonal injury is increasing. The data suggest that elevation in proteins associated with mitochondria and oxidative phosphorylation closely accompanies the acceleration in WD of the distal compartments of the motor neuron following axotomy. Specifically, there was a shared increase in protein expression associated with Complex I and IV of the OXPHOS pathways in both muscle and nerve. An increase in both the activity and levels of Complex I was confirmed in muscle. We also demonstrate that inhibition of Complex I prevents the axotomy related rise in ROS and protects the axon from degeneration. Finally, using the *ex-vivo* model of neuronal injury, we demonstrate that up-regulating basal OXPHOS levels ablates the delay in WD observed in neonatal mice. These findings imply a regulatory role for mitochondria in synaptic maintenance and degeneration. The data also provide evidence for a dynamically evolving postnatal proteome, including altered levels of mitochondrial proteins that can influence sensitivity to triggers of synaptic and axonal degeneration.

### 4.1 Implications of the evolving postnatal proteome for neuromuscular disease

The findings we present in this study demonstrate that a broad range of molecular pathways evolve steadily and dynamically over postnatal development. It is therefore likely that a broad array of molecular pathways, including those potentially impacting upon pathogenic cascades, could be markedly influenced by the age of the individual. This immediately invites consideration into the family of neuromuscular diseases arising in early childhood, including: spinal muscular atrophy, Duchenne's muscular dystrophy, Charcot-Marie-Tooth disease and spinal bulbar muscular atrophy. There are clearly important implications both for the use of animal models to research these diseases, and for the prognosis and treatment of patients.

Mouse models of a variety of neuromuscular disorders tend to manifest during the first post-natal month. The is certainly true of those models mimicking pediatric neuromuscular disorders (Bowerman et al., 2012; Le et al., 2005), but is also evident in spontaneous mutants with peripheral neuropathies (Hedlund et al., 2010; Brown et al., 1995) and models mimicking adult onset disorders (Schmalbruch et al., 1991; Xu et al., 1993). Such models are commonly used to understand the molecular and structural events which occur during the disease time course. Whilst this is clearly of great importance, it is also important to consider how the evolving proteome impact upon the pathological processes. Some pertinent examples of this arise from the SMA research field. This includes a marked difference in the severity in pathology when *Smn*, the disease gene, is turned off in adulthood compared to in neonates (Kariya et al., 2014) and a change in the severity and pattern of selective vulnerability between SMA mouse models which manifest at different ages (Bowerman et al., 2012; Le et al., 2005; Murray et al., 2008). The data we present here suggest that the differences in the manifestation of neuromuscular pathology observed between these mouse models is due to the evolving proteome occurring during the time period. Indeed, several proteins which are known to impact upon the pathological mechanisms implicated in neuromuscular pathology in SMA and other motor neuron diseases were found to be altered during the postnatal period. This includes proteins such as IGF2 which has been shown to be an important modifier in motor neuron disease (Allodi et al., 2016; Hedlund et al., 2010).

It becomes increasing apparent that it will be of great importance to understand how the timing of murine postnatal development relates to human postnatal development. Mice clearly have a different lifespan, and are born with considerably different motor abilities, and potentially at a different stage of neuromuscular maturity. The morphological events occurring in the first postnatal month in the mouse have been extensively characterized, but the lack of available tissue makes it very difficult to know if and when these morphological changes occur into humans. In mice, axons of the ventral ramus appear to grow out from the spinal cord at around E12.5, reach the dorsal and ventral muscle masses at around E13.5, and initial nerve muscle contacts are formed at around E14.5 (Hurren et al., 2015). Acetylcholine receptor clusters are not observed until around E14, becoming numerous at around E15 (Hurren et al., 2015). This suggests formation of neuromuscular contacts occurs at around 60 to 70% of the way through gestation. Detail on the equivalent process in human is inevitably more sparse, but clusters of acetylcholine receptors and axon bundles have been observed at around 10 gestational weeks, with plaque like endplate structure visible at 14 gestational weeks (Martinez-Hernandez et al., 2013). It has also been suggested that mono-innervated endplates could be observed at 14 gestational weeks (Martinez-Hernandez et al., 2013). Human NMJ formation therefore likely occurs at around 30% of the way through gestation. Based on these figures, it would appear that the initial formation of NMJs occurs at a comparably earlier time point in humans than it does in mice. It is therefore tempting to assume that humans are born with a comparatively more mature neuromuscular system although clearly the details of human neuromuscular maturation requires further investigation. However, the current profiling of molecular events in mice will be of fundamental importance for future comparison with human data.

### 4.2 The role of mitochondria in WD

Functional annotation analysis of proteins exhibiting a steady up-regulation over the P12-P24 period revealed a striking enrichment for the processes of OXPHOS and associated mitochondrial functions, in both the nerve and muscle. Strikingly, pathway analysis of proteomic alterations occurring in both the nerve and the muscle further suggested a conserved escalation in mitochondrial OXPHOS activity and redox sensitivity pathways. Taken together, these findings invite consideration of both how this might relate to the progressive acceleration in the rate of WD, and how this information may impact upon our understanding of the mechanisms of synaptic and axonal degeneration.

The implication of mitochondria in the process of WD is certainly not novel, although there is controversy as to the extent to which they are required for the process of WD. Mitochondrial swelling during WD has been noted in early ultra-structural studies of degenerating nerves (Vial, 1958). It has been shown that over-expression of the mitochondrially localized form of Nicotinamide mononucleotide adenylyltransferase, Nmnat3, can protect axons from injury in both mouse and drosophila models (Avery et al., 2012; Berger et al., 2005; Yahata et al., 2009). The specifics of the involvement of mitochondria in WD remain highly debated. It is clear that axon injury results in a reduction in mitochondrial motility, and that this sudden reduction in mitochondrial motility observed in wildtype axons following axotomy, is markedly suppressed in Wld^s^ axons (Avery et al., 2012). However it has also been shown that there is also reduced mitochondrial motility in axons from *Sarm1* deficient mice, in which the axons show a similar Wld^s^ like protection from axon degeneration (Summers et al., 2014). This suggests that a reduction in mitochondrial motility is not necessary for axon degeneration. A number of other cellular events which are closely linked with mitochondria have been shown to have an important effect upon axon degeneration. This includes energy depletion, as evidenced by a reduction in NAD and ATP levels, a rise in calcium levels and increased production of reactive oxygen species (Park et al., 2013). Indeed it has been suggested that a key event in axon degeneration is the opening of the mitochondrial permeability transitioning pore, which results in the observed swelling of the mitochondria and the release of ROS and increase in calcium levels (Barrientos et al., 2011; Villegas et al., 2014; Calixto et al., 2012). The specifics of the relationships between the individual factors which have been shown to influence axon degeneration remain to be defined. However, from this work, an important role for mitochondria is evident. For this reason, our observed increase in mitochondrial protein expression is highly likely to be associated with the acceleration in the rate of WD.

How then may the progressive increase in mitochondrial proteins observed in neonatal mice lead to the progressive increase in the rate of WD? The proteomic analysis points to a specific enrichment for proteins pertaining to Complex I of the electron transport chain. Complex I has been shown to be the rate limiting step in energy production at the nerve terminal, a major source of protons for ATP synthesis and therefore a major source of ROS production (Giachin et al., 2016; Telford et al., 2009). ROS levels rise rapidly after axon injury and inhibition of ROS can delay axon degeneration (O'Donnell et al., 2013). Indeed, here we show that the rise in ROS following injury in prevented by application of a Complex I inhibitor, and this correlated with protection of the axon. This rise is ROS levels is not observed in *Wld*^*s*^ mice, thus presenting a possible mechanism of action (O'Donnell et al., 2013). Furthermore, although the rise in ROS is still observed in *Sarm1*^*−/−*^ axons, they appear to be somehow resistant to oxidative damage caused by ROS. This suggests that Sarm1 may mediate the damage caused by ROS, and loss of Sarm1 leads to a delayed axon degeneration as the cell is protected from damage caused by ROS (Summers et al., 2014). Given that Complex I is a major source of ROS production (Giachin et al., 2016; Telford et al., 2009), and inhibition of Complex I is protective to axons, it seems reasonable to speculate that the increase in Complex I activity between P12 and P24 can account for the acceleration in the rate of WD over this same time period. Similarly, the lower level of Complex I activity in neonatal nerve, results in a slower production of ROS after nerve injury, and therefore a slower rate of WD. Previous work has also shown that the opening of the MPTP induces a conformational change in Complex I, which leads to a rapid increase in ROS production and release (Batandier et al., 2004). It is therefore possible that the progressive increase in Complex I levels between P12 and P24 lead to increased ROS production following MPTP opening during WD. In future work, it would be interesting to address the basal and induced levels of ROS during axon degeneration in neonatal nerve.

## 5 Conclusions

Quantitative analysis revealed that the neuromuscular proteome changes between the ages of P12 and P24, dynamically altering the state of a broad range of molecular networks, most significantly in those involved in mitochondrial bioenergetics. Based on these findings, we determined that pharmacologically up-regulating basal OXPHOS activity at P12, an age characterized both *in vivo* and *ex-vivo* by a delay in the rate of synaptic degeneration, results in significantly increased sensitivity of NMJ to axotomy. This suggests that basal levels of mitochondrial OXPHOS activity may pre-condition the motor neuron to degenerate more rapidly in the event of traumatic injury, perhaps thereby facilitating axonal regeneration. We show than inhibition of Complex I can prevent the axotomy related increase in ROS and that this correlates with axon protection. Elucidating the mechanism by which mitochondrial bioenergetics contributes to the injury-induced molecular response should enhance understanding of motor neuron degeneration. In a broader context, alterations in protein expression across early postnatal development may influence the particular vulnerability of the motor neuron and skeletal muscle in neuromuscular diseases of early childhood.

The following are the supplementary data related to this article.

Supplementary material Image 1
